# Isolated adrenocorticotropic hormone deficiency associated with sintilimab therapy in a patient with advanced lung adenocarcinoma: a case report and literature review

**DOI:** 10.1186/s12902-022-01151-y

**Published:** 2022-09-24

**Authors:** Si-Hong Lin, Ao Zhang, Lu-Zhen Li, Liang-Chen Zhao, Le-Xia Wu, Can-Tu Fang

**Affiliations:** 1 Zhongshan Affiliated Hospital, Guangzhou University of Chinese Medicine, 23 Kangxin Road, Zhongshan, 528400 Guangdong China; 2 Shunde Hospital, Guangzhou University of Chinese Medicine, Foshan, China

**Keywords:** Sintilimab, Programmed cell death protein 1, Isolated ACTH deficiency, Corticosteroid replacement therapy, Case reports

## Abstract

**Background:**

Several immune checkpoint inhibitors have been implemented for cancer treatment which have shown some degree of antitumor effcacy, while immune-related adverse events (irAEs) that affect multiple organ functions ensue which obviously should not be neglected. Though less common than other kinds of irAEs, Immune checkpoint inhibitors (ICIs) related Isolated ACTH deficiency (IAD) may cause long-term damage to pituitary-adrenal axis. Several case reports are available about IAD during anti-PD-1 therapy. We report the first case of immune checkpoint inhibitor-induced IAD following 3 month of sintilimab therapy.

**Case presentation:**

A 66-year-old Chinese man was diagnosed with stage IIIB lung adenocarcinoma with involving ipsilateral intrapulmonary and hilar lymph node metastasis. After 3 months of combination therapy of nedaplatin, pemetrexed and sintilimab, the patient presented with general fatigue, nausea and vomiting. Laboratory investigation at admission revealed hyponatremia and hypokalemia. Further investigation revealed adrenocorticotropic hormone and cortisol levels were far below than normal limits. His other pituitary hormone levels were normal, except for mild elevation of follicle stimulating hormone and estradiol. Cranic magnetic resonance imaging showed a normal pituitary gland. Isolated adrenocorticotropic hormone deficiency was diagnosed, and corticosteroid replacement therapy was administered, leading to a significant improvement of his symptoms while ACTH level maintaining low level.

**Conclusions:**

Our patient developed isolated ACTH deficiency during combination cancer treatment with chemotherapy and sintilimab. Although isolated ACTH deficiency due to anti-PD-1 including sintilimab therapy is rare occurrence, it can often cause severe clinical symptoms. Its diagnosis basically relies on clinical symptoms and endocrinological examination. Unlike traditional hypophysitis diagnosed by cranial MRI, pituitary MRI of IAD due to anti-PD-1 often indicates normal pituitary gland implying that over-reliance on imaging findings is not recommended. Even if clinical symptoms have relieved after corticosteroid replacement therapy was commenced, low levels of ACTH or cortisol could maintain for a long period which highlights the need for long term corticosteroid therapy. The purpose of the current report was to provide increased awareness of early detection and therapy of IAD.

## Background

As an anti-tumor treatment approach, immunotherapy is now making progress. Different from conventional cancer treatments including radiotherapy, chemotherapy and targeted agents, immunotherapy is not designed to act directly on tumors but to harness the patient’s own immune systerm to fight cancer [1] based on the concept of reactivation of T cells can block tumor cells from escaping the immune system. As a result, by unbalancing the immune system, these immune checkpoint inhibitors (ICIs) could generate dysimmune toxicities, called immune-related adverse events (IrAEs) that mainly involve diverse organs or tissues due to off-target effect since ICIs action mechanism relies on the inhibition of the physiological brake of immune activation [2, 3]. Hypothyroidism/hyperthyroidism and hypophysitis are confirmed as the most frequently occurring endocrine irAEs [4]. And Pituitary, thyroid, and adrenal glands are endocrine organs typically affected by ICIs treatment following the ICIs use [5].

Regarded as a subtype of hypophysitis [6], IAD, which is characterized by secondary adrenal insufficiency, is a rare but potentially lethal adverse effect of ICIs. It is featured with low or absent cortisol production, normal secretion of pituitary hormones other than ACTH and free from structural pituitary defect. Morning serum cortisol below 3 µg/dl are virtually diagnostic for adrenal insufficiency. If accompanied by low level of ACTH at the same time, however is suggestive of secondary adrenal insufficiency. Being the first to describe the prevalence of IAD among patients treated with multiple types of ICI, one large cohort defined that the risk factors for developing IAD include female gender and treatment with multiple ICI types. Reportedly, patients with IAD usually manifests as fatigue, anorexia, gastrointestinal discomfort and rarer hypotension [7].

Glucocorticoid replacement therapy has proven effective. Nevertheless, its monitoring essentially relies on clinical judgment since no laboratory parameter is fully reliable. Furthermore, a potential negative effect of high glucocorticoid doses on the efficacy of checkpoint inhibitors after an irAE has been reported which highlights the need for physicians to be cautious of the appropriate dose. But these findings have potential implications for the management of other irAEs [8].

As a fully human IgG4 monoclonal antibody, Sintilimab, binds toprogrammed cell death receptor-1 (PD-1), thereby blocking the interaction of PD-1 with its ligands (PD-L1 and PL-L2) and consequently helping to restore the endogenous antitumour T-cell response. It has been approved in CHINA for the treatment of classical Hodgkin’s lymphoma in patients who have relapsed or are refractory after ≥ 2 lines of systemic chemotherapy [9]. Data from clinical tral prove that Sintilimab also shows considerable anti-tumor effects in non-small cell lung cancer, digestive system tumors, etc. [6, 10]. Common sintilimab-associated irAEs include pyrexia, hypothyroidism, rash, pneumonitis, fatigue and decreased platelet count [11, 12]. Up till now, isolated ACTH deficiency due to sintilimab therapy has not been reported as far as we concerned.

We herein reprt on a patient with stage IIIB lung adenocarcinoma who developed IAD during Sintilimab therapy. In addition, previously cases of IAD in association with anti-PD-1 treatment are reviewed.

## Case presentation

**Fig. 1:**
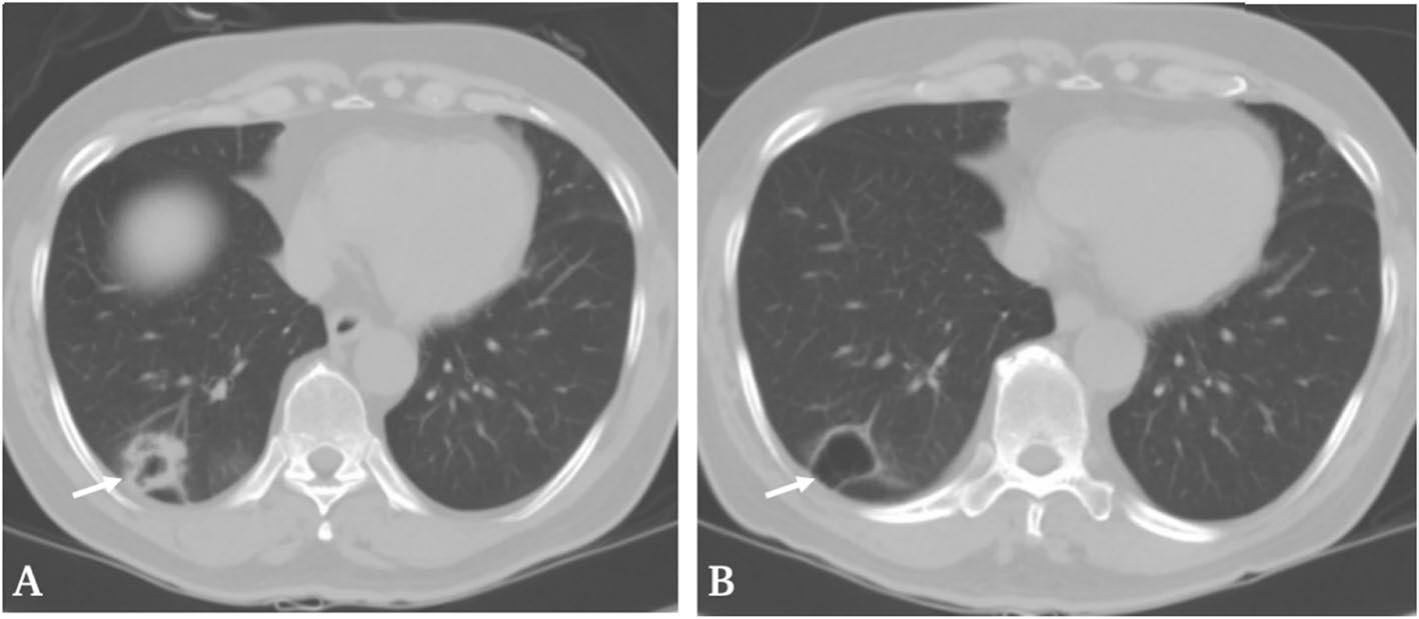
Chest computed tomography (CT) scans. **A** chest CT performed in June 2020 showing a 2.5 cm tumor in the low lobe of the right lung (white arrow). **B** chest CT performed in August 2020 showing a slight reduction in the diameter (2.4 cm) and marked reduction of solid component of the lung tumor (white arrow)

A 66-year-old man was admitted to our hospital in September 2020 because of general fatigue, nausea and vomiting. He had a history of cervical spondylotic myelopathy and cerebral infarction. His brother had a history of bladder cancer. The patient had smoked 40 cigarettes per day for 50 years but did not have a drinking habit. In May 2020, he was diagnosed with lung adenocarcinoma involving ipsilateral intrapulmonary and hilar lymph node metastasis (cT4N2M0 stage IIIB) and PD-L1 test showed high expression with 85%TPS. ALK, EGFR, ERBB2, MET, ROS1 genes mutations were negative. Initially, the patient received 1 course of chemotherapy with intravenous nedaplatin and pemetrexed (the dose of treatment was unknown). Subsequently, the patient came to our hospital for treatment. After a multidisciplinary discussion, he received 3 courses of chemotherapy with intravenous nedaplatin, pemetrexed and sintilimab (totals of 2550 mg, 270 mg and 600 mg, respectively) from June 2020 to August 2020. This treatment regimen had effectively controlled his LAC with a Response Evaluation Criteria in Solid Tumors (RECIST) classification of partial response according to lung computed tomography (CT) performed in August 2020 (Fig. 1B).

In August 2020 the patient’s body length, body weight, body temperature, blood pressure, pulse rate and respiratory rate were 163.5 cm, 61.7 kg, 36.3℃, 148/90 mmHg, 121beats per minute, 20 breaths per minute, respectively. His serum electrolyte levels (sodium, 142.4 mmol/L; potassium, 4.04 mmol/L; cholride, 108.3 mmol/L) were within normal ranges. Basal endocrinological data showed normal levels of serum cortisol (379.50 nmol/L) and plasma ACTH (12.56 pmol/L). However, the patient developed general fatigue, nausea and vomiting in September 2020 before the 4th (next) round of therapy in September 2020.

At admission, the patient had a clear consciousness without complaints of headache, dizziness, chest pain, abdominal pain, abdominal bloating, diarrhea and physical examination revealed his body temperature was 36.5 °C, blood pressure was 130/75 mmHg, pulse rate was 98 beats per minute, and respiratory rates was 18 breaths per minute. No heart murmur and chest rales, vitiligo, rash, skin pigmentation, peripheral edema were detected. A blood analysis revealed hyponatremia(serum sodium, 135.8 mmol/L), hypokalemia (serum potassium, 3.82 mmol/L) and low levels of RBC (3.94 × 10^12/L), hemoglobin (122 g/L) and HCT (23%) (Table 1).

**Table 1:** Laboratory Findings on Admission (September 2020)

Laboratory data	Clinical values	Reference ranges
Red blood cells	3.94 × 10^12/L	4.30–5.80
Hemoglobin	122 g/L	130–175
Hematocrit	36%	40–50
White blood cells	4.40 × 10^9/L	3.50–9.50
Eosinophil	0.34 × 10^9/L	0.02–0.52
Eosinophil ratio(%)	7.70%	0.4–8.0
Platelets	329 × 10^9/L	125–350
Alanine aminotransferase	49U/L	9-50U/L
Aspartate aminotransferase	41U/L	15-40U/L
Total protein	64.6 g/L	65.0–85.0
Albumin	38.0 g/L	40.0–55.0
Immunoglobulin	26.6 g/L	20.0–40.0
Creatinine	104umol/L	57–111
Casual plasma glucose	5.56 mmol/L	3.89–6.11
Potassium	3.82 mmol/L	3.50–5.30
Sodium	135.8 mmol/L	137.0–147.0
Chloride	97.9 mmol/L	99.0–110.0
Calcium	2.05 mmol/L	2.11–2.52

In view of the patient’s medical history, brain magnetic resonance imaging (MRI) and cervical vertebra computed tomography (CT) were immediately performed. But they showed no evidence of acute exacerbation of cervical spondylotic myelopathy, acute cerebral infarction (ACI) or LAC brain metastasis. The patient was symptomatically treated with drip infusions and oral agents. However, his symptoms didn’t resolved.

**Fig. 2:**
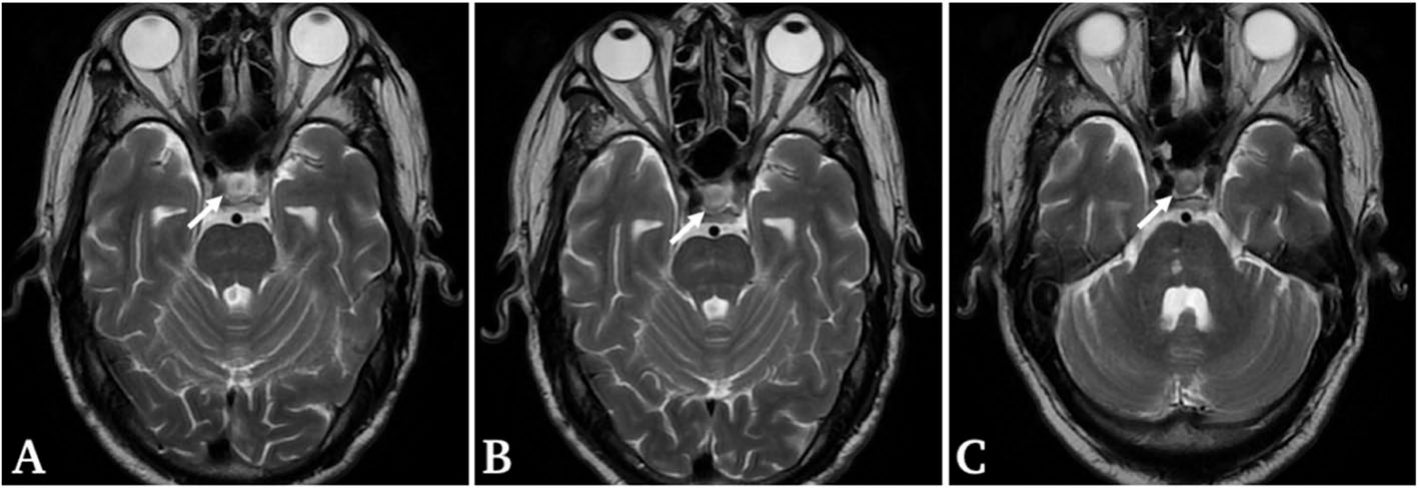
Magnetic resonance imaging (MRI) scans. **A** Brain MRI scans obtained before the diagnosis of isolated adrenocorticotropin deficiency (August 2020). **B**, **C** Brain MRI scans obtained at and after the diagnosis of IAD, respectively (September and November 2020). **A-C** all MRI scans showing a normal size of pituitary gland and no obvious enhancement was observed (white arrows)

On day 6 of admission, laboratory tests revealed plasma ACTH and serum cortisol levels were far below than normal limits, and mild elevation of follicle stimulating hormone (36.85mIU/mL) and estradiol (151.7 pmol/L). The patient’s other pituitary hormone levels were normal (Table 2). Brain magnetic resonance imaging scans revealed neither space-occupying lesions nor heterogeneous enhancement of the pituitary gland (Fig. 2B).

**Table 2:** Basal endocrinological data of present case (September 2020)

	Clinical values	Reference ranges
Free triiodothyronine	4.91 pmol/L	3.5–6.5
Free thyroxine	17.41 pmol/L	11.5–22.7
Thyroid-stimulating hormone	0.89uIU/mL	0.51–6.27
Prolactin	20.8uIU/mL	43–375
Follicle stimulating hormone	36.85mIU/mL	1.4–18.1
Luteinizing hormone	22.02mIU/mL	3.1–34.6
Testosterone	23.7 nmol/L	3.02–27.07
Estradiol	151.7 pmol/L	0–146.1
ACTH(4 pm)	< 0.22 pmol/L	1.6–13.9
Cortisol(4 pm)	8.87 nmol/L	68.20–327.00
ACTH(0am)	< 0.22 pmol/L	1.6–13.9
Cortisol(0am)	9.89 nmol/L	6-10am|133.00–537.00 4-8 pm|68.20–327.00
ACTH(8am)	< 0.22 pmol/L	1.6–13.9
Cortisol(8am)	10.42 nmol/L	133.00–537.00

Since the patient was suspected having Isolated ACTH deficiency (IAD), corticosteroid replacement therapy was commenced at day 16 of admission. 100 mg methylprednisolone was injected intramuscularly from day 16 to 18, and was reduced to 80 mg on day 19 and maintained for 6 days, and reduced to 60 mg on day 25 for 3 days. The patient experienced significant improvements in general fatigue, nausea and vomiting. So he was discharged on 27th day of admission and received 40 mg oral agents for maintenance treatment for 7 days.

However, the patient developed general fatigue, nausea and vomiting again 9 days after he was discharged, when he discontinued corticosteroid therapy for only 2 days. At admission, physical examination revealed his body height was 163.5 cm, body weight was 61.7 kg, body temperature was 36.8 °C, blood pressure was 120/85 mmHg, pulse rate was 106 beats per minute, and respiratory rates was 20 breaths per minute. His blood analysis again revealed hyponatremia (serum sodium, 133.6 mmol/L) (Table 3). Immune related IAD was highly suspected so corticosteroid replacement therapy was immediately commenced at the admission. On day 2 of the admission, endocrinological data indicated low level of plasma ACTH, and high level of follicle stimulating hormone (29.44mIU/mL), while serum cortisol was within normal limit (Table 4). brain MRI study showed no enlargement of the pituitary gland (Fig. 2C). 100 mg methylprednisolone was injected intramuscularly from day 1 to day 6. On day 7, the dose was reduced to 80 mg per day and maintained for 6 days. 60 mg/d was maintained for 5 days and 40 mg/d for 10 days. The patient’s symptoms resolved and was discharged on 20th day of admission. But unfortunately, we failed to follow up the patient.

**Table 3:** Laboratory Findings on Admission (October 2020)

	Clinical values	Reference ranges
Red blood cells	4.55 × 10^12/L	4.30–5.80
Hemoglobin	136 g/L	130–175
Hematocrit	43%	40–50
White blood cells	6.99 × 10^9/L	3.50–9.50
Eosinophil	1.40 × 10^9/L	0.02–0.52
Eosinophil ratio(%)	1.40%	0.4–8.0
Platelets	270 × 10^9/L	125–350
Alanine aminotransferase	22U/L	9-50U/L
Aspartate aminotransferase	24U/L	15-40U/L
Total protein	67.3 g/L	65.0–85.0
Albumin	37.9 g/L	40.0–55.0
Immunoglobulin	29.4 g/L	20.0–40.0
Creatinine	87umol/L	57–111
Casual plasma glucose	3.49 mmol/L	3.89–6.11
C-reactive protein	143.40 mg/L	0–3
Potassium	4.28 mmol/L	3.50–5.30
Sodium	133.6 mmol/L	137.0–147.0
Chloride	93.6 mmol/L	99.0–110.0
Calcium	2.01 mmol/L	2.11–2.52

**Table 4:** Basal endocrinological data of present case(October 2020)

	Clinical values	Reference ranges
Free triiodothyronine	3.92 pmol/L	3.5–6.5
Free thyroxine	27.04 pmol/L	11.5–22.7
Thyroid-stimulating hormone	0.88uIU/mL	0.51–6.27
Prolactin	32.8uIU/mL	43–375
Follicle stimulating hormone	29.44mIU/mL	1.4–18.1
Luteinizing hormone	25.30mIU/mL	3.1–34.6
Testosterone	15.3 nmol/L	3.02–27.07
Estradiol	145.1 pmol/L	0–146.1
ACTH(8am)	< 0.22 pmol/L	1.6–13.9
Cortisol(8am)	241.10 nmol/L	133.00–537.00

## Discussion and conclusions

We here report the first case of isolated ACTH deficiency (IAD) associated with sintilimab therapy. The patient was a 66-year-old Chinese male who was diagnosed with LAC (cT4N2M0 Stage IIIB) in May 2020. PD-L1 test showed high expression with 85%TPS. ALK, EGFR, ERBB2, MET, ROS1 genes mutations were negative. He developed Isolated ACTH deficiency and manifested predominantly as general fatigue, nausea and vomiting following three months of sintilimab therapy combined with pemetrexed and nedaplatin. Corticosteroid replacement therapy was effective and the patient’s condition rapidly improved.

IAD is considered as a subtype of hypophysitis [6], which is characterized by secondary adrenal insufficiency. Endocrinological examinations show low values of serum cortisol and normal secretion of pituitary hormones other than ACTH. In contrast of conventional hypophysitis, MRI scans usually show no structural defects in the pituitary gland. The main symptoms of IAD include fatigue, anorexia, gastrointestinal discomfort and rarer hypotension. According to some reports, women are at higher risk of IAD than men, and the combined application of ICIs is also a risk factor.A systematic review [13] which identified of a total of 60 IAD cases revealed that melanoma was the tumor most frequently reported and nivolumab in monotherapy was the most commonly associated ICIs therapy. A retrospective study found that no patients with Ipilimumab monotherapy generated IAD whereas combination of PD-1/PD-L1 inhibitors and Ipilimumab result in higher risk for developing IAD compared to only one of the drugs which is consistent with former study [7]. A large sample case series study discovered that anti-PD-1-induced group had more IAD patients than anti-CTLA-4 induced group and longer median duration of anti-PD-1 therapy usage may be responsible for the difference [14]. Moreover, deficiencies in other pituitary axes other than pituitary-adrenal axis were observed more often in the ipilimumab monotherapy which may also account for the difference [15]. Given the relatively high incidence of PD-1-induced IAD, it is necessary to collate the reported cases of anti-PD-1-induced IAD. Table 5 summarizes the characteristics of the patients who exhibited IAD related to cancer treatment with anti-PD-1.

**Table 5:** Summary of Reported Patients who Exhibited Isolated Adrenocorticotropin Deficiency (IAD) during Cancer Treatment with anti-PD-1

Case	Age/sex	Target cancer	Type of anti PD-1	Regimen	Time to onset of IAD/course	Major symptom-s at IAD onset	MRI of pituitary gland	Hyponatr-emia	Eosinopgi-lia	Treatmen-t	Ref
1	79/M	Lung adenocarcinoma	Nivo	NA	8.5 M/20	AL, nausea, Difficult waking	NP	( +)	(-)	HC	[16]
2	71/M	Renal cell carcinoma (multiple lung metastases)	Nivo	3 mg/kg/2w	6 M/14	AL,GM, Mild conscious-ness disturban-ce	NP	( +)	( +)	HC,PDN	[17]
3	39/M	metastatic malignant melanoma	Nivo	3 mg/kg/2w	9 M/13	GM	NP	(-)	( +)	HC	[18]
4	53/M	advanced melanoma	Nivo	3 mg/kg/2w	14 W/NA	GF, muscle pain, vomiting	NP	(-)	( +)	HC	[19]
5	72/M	advanced melanoma	Nivo	3 mg/kg/2w	30 W/NA	AL, GF, vomiting	NP	( +)	NA	HC	[19]
6	76/F	Metastatic melanoma	Nivo	2 mg/kg/3w	NA/9	AL, bradykine-sia	NA	( +)	NA	cortisol	[20]
7	75/M	Lung adenocarcinoma	Nivo	3 mg/kg/2w	23 W/9	AL, Progressi-ve fatigue	NP	( +)	(-)	HC	[21]
8	61/M	Squamous cell carcinoma	Nivo	3 mg/kg/2w	15 W/8	AL, GM	NP	( +)	(-)	HC	[22]
9	63/F	Lung adenocarcinoma	Nivo	3 mg/kg/2w	8 M/17	Anorexia,fatigue, myalgia, Difficult walking	slightly thickened	( +)	NA	HC	[23]
10	73/M	Squamous cell carcinoma	Nivo	3 mg/kg/2w	5 M/NA	GF, arthralgia	NP	NA	NA	HC	[24]
11	74/F	Renal Cell Carcinoma( pulmonary metastasis)	Nivo	3 mg/kg/2w	NA/5	AL, GF, nausea	NP	( +)	( +)	HC	[25]
12	58/M	Malignant Melanoma( liver metastasis)	Nivo	3 mg/kg/2w	8 M/NA	AL, fatigue, weakness	NP	(-)	NA	HC	[26]
13	52/F	Breast cancer(lung and liver metastasis)	Nivo	0.36 mg/kg/2w	6 M/NA	GF	NP	NA	NA	HC	[27]
14	70/M	advanced urothelial carcinomar (of the left kidney)	Nivo	3 mg/kg/2w	NA/9	anorexia,general weakness, nausea	NP	( +)	NA	HC	[28]
15	65/F	metastatic cecal cancer	Keytruda	NA(200 mg/NA)	7 W/2	severe fatigue	NP	NA	NA	HC	[29]
16	85/F	Advanced squamous cell lung carcinoma	Keytruda	200 mg/3w	NA/8	AL,GF	higher intensity on T2-weighted imaging	( +)	(-)	HC	[30]
17	59/M	Non-small-cell Lung Cancer(adrenal metastasis)	Keytruda	200 mg/3w	7 M/5	anorexia, fatigue, slight fever	NP	( +)	NA	HC	[31]
Our case	66/M	Lung adenocarcinoma	Sintilimab	200 mg/3w	3 M/3	GF, nausea, vomiting	NP	( +)	(-)	PDN	/

*M* Male, *F* Female, *AL* Appetite loss, *GM* General malaise, *GF* General fatigue, *NP* Nothing particular, *NA* Data not available, *HC* Hycrocortisol, *PDN* Prednisone

These patients included women and men who exhibited IAD after seven weeks to nine months of the initiation of PD-1 inhibitors [16–31]. In the present case, our patient developed IAD following three months of sintilimab therapy which was consistent with the previous belief that anti-PD-1 o/PD-L1 therapy-induced hypophysitis appeared roughly within three to five months. It was reported that the endocrinologic examination may show a decline of some indicators within the normal range before the symptoms of IAD appeared, indicating the possibility of the trend towards pituitary and adrenal dysfunction. While in some other cases, patients’ plasma ACTH and serum cortisol had already showed a reduced level than normal before they experienced some relevant physical discomforts, which means that pituitary and adrenal disorder could have already existed [32]. Therefore, pituitary and adrenal hormone levels should be monitored before and during the use of PD-1 inhibitors, which may significantly contribute to the early detection and treatment of PD-1-induced secondary adrenal insufficiency. Furthermore, delayed presentation of Isolated ACTH deficiency after months of PD-1 inhibitors withdrawal dose not seem to be so infrequent. Shiva Shrotriya described a patient with NSCLC developed Isolated ACTH deficiency seven months after completing nivolumab therapy [33]. Satoshi Yamagata presented a case that a patient developed IAD four months after pembrolizumab discontinuation [31]. The mechanism of this has not been clarified, but it may be related to PD-1 occupancy. Experimental studies have noted that after a single infusion of nivolumab, the molecules can be found to be bound for over 2 months [34]. As for Sintilimab, a sustained PD-1 receptor occupancy of more than 95% for up to 4 weeks was observed after a single infusion intravenously [6]. This brings into focus that the need for continuously monitoring the pituitary and adrenal hormone levels. But it still remains unknown for how long.

Although IAD usually manifests as some non-specific symptoms, including fatigue, loss of appetite, nausea, vomiting, general malaise, myalgia, disturbance of consciousness, fever, hyponatremia, hypotension, hypoglycemia, eosinophilia. But we have observed that the main symptoms are concentrated on fatigue and loss of appetite, regardless of which kind of anti-PD-1 therapy was commenced, reminding that when patients using anti-PD-1 therapy exhibit sudden unexplained fatigue and loss of appetite, clinicians should complete relevant laboratory tests as soon as possible for early detection of ICIs-related IAD. Our patient generated grade 3 Isolated ACTH deficiency which manifested as general fatigue, nausea, vomiting and Hyponatremia following 3-month Sintilimab therapy, indicating that Sintilimab, same as other PD-1 inhibitors, could cause immune-related IAD. ACTH deficiency can result in a decrease in glucocorticoids, which in turn inhibits the secretion of vasopressin. Severe hypotonic hyponatremua could arise from vasopressin hypersecretion-like pathological conditions in cases of ACTH deficiency [33]. Among cases summarized above (Table 2), 11 out 18 (67%) patients exhibited hyponatremia, and 4 out of 18 patients (22%) exhibited eosinophilia as they developed IAD. To our knowledge, individual cases showed that hyponatremia and eosinophilia even appeared earlier than secondary adrenal deficiency, implying potential association between them and the incidence of IAD. Consequently, the serum sodium level is regarded to be useful in predicting progression to isolated ACTH deficiency and eosinophilia was also believed to be an early predictor of adrenal insufficiency [16, 35, 36]. We also found that patients with concomitant hyponatremia are more common as compared to those with eosinophilia. Our patient was also accompanied by hyponatremia on admission, and there was a trend of progressive decline during hospitalization. But it finally showed improvement after therapy. This alerted to us a clinically important message that continuous monitoring of serum sodium levels and eosinophil counts may contribute to early detection of isolated ACTH deficiency and could be one of the indicators of efficacy evaluation simultaneously.

A radiological follow-up study reported that 181 cases (88%) of 204 patients with autoimmune hypophysitis showed initial swelling of the pituitary gland [37]. Unlike traditional hypophysitis diagnosed by cranial MRI, although the test results suggest pituitary dysfunction, the cranial MRI of patients with IAD often indicates normal size of pituitary gland. All patients showed pituitary-adrenal axis dysfunction in the cases reviewed, while only 2 out of 18 (11%) patients showed abnormality of pituitary on MRI. Perhaps the MRI may be less sensitive to hypophysitis in relation to anti-PD1 therapy [36]. Longitudinal case cohorts have revealed that 88 of 112 cases (78.6%) with ipilimumab-induced hypopituitarism exhibited radiographic enlargement of the pituitary gland [38], suggesting that morphologic changes in the pituitary may be less severe in nivolumab-induced hypophysitis patients than that in ipilimumab-induced hypophysitis patients. It is worth noting that even if the MRI was indicative of abnormality of pituitary gland, no evident extent was presented, suggesting the significance of pre- and post-contrast of cranial MRI. Moreover, pituitary enlargement of PD-1 treatment-related IAD was observed to be reversible [23]. Though MRI findings indicative of pituitary restored to its normal size and shape, it could still be suggestive of remaining a state of secondary adrenal dysfunction within some patients through endocrine tests.This implies that MRI imaging in the diagnosis of IAD is somehow helpful while limited in most of our cases, suggesting that over-reliance of imaging findings probably results in misdiagnosis and delayed treatment of treatable irAEs based on the decoy of normal MRI presentation of pituitary imaging which should be avoided.

Corticosteroid replacement therapy has proven to be effective. However, its monitoring essentially relies on clinical judgment as no laboratory parameter is fully reliable. Our patient discontinued 18 days after the glucocorticoid treatment was commenced during the first hospitalization, and was re-admitted to the hospital for the presentation with same symptoms 2 days after the discontinuation. 100 mg methylprednisolone was injected intramusculary on the day of second admission. It turned out that cortisol level was in the normal range, while there continued to be significantly lower than normal in ACTH levels, which is in accordance with previous reports that ACTH remained lower than normal level suggesting patients remaining the state of Isolated ACTH deficiency in spite of the evident relief of the symptoms after treatment. The recovery of the pituitary-thyroid axis has been reported in 37–85% of cases in irAEs, while dysfunction of the pituitary-adrenal axis is unlikely to recover in such cases [39]. That is to say, it could cause long-term damage to pituitary-adrenal axis, thus requiring long-term replacement of hydrocortisone in most patients confirmed with IAD, which has a positive effect on continuing immunotherapy [31].

Although IAD may lead to temporary suspension of ICI-based cancer therapy, it seems that if IAD is appropriately controlled, ICI therapy could be continued to maximize its benefit. A prospective study [40] revealed that, associated with better OS for both non-small cell lung carcinoma and malignant melanoma when patients are treated with physiological doses of hydrocortisone, the development of pituitary-irAEs including IAD, might be a potential predictor of better prognosis. However, the mechanism of such association still remains unclear. This may be related to the continuation of ICIs treatment after appropriate corticosteroid therapy provided symptomatic relief of pituitary-irAEs. Among cases summarized above (Table 5), we found that some patients (case1,2,15) benefit from continuing ICIs treatment after relief of PD-1-induced IAD. While in some other cases (case4,5,6,8), restarting anti-PD-1 treatment together with corticosteroid therapy is also beneficial. Currently, studies on the relationship between ICIs related Isolated ACTH deficiency and efficiency of ICIs treatments are still limited. Given that patients who develop pituitary-irAEs while on ICIs therapy are reported to fare better than those who do not, it seems critically important to ensure that IAD is early detected as well as appropriately controlled, and immunotherapy is continued as long as possible. Regrettably, our patient failed to be followed up after second hospitalization due to personal reason.

In summary, manifesting as diverse and non-specific symptoms, isolated ACTH deficiency could be easily misdiagnosed. As a result, thorough inspections and continuous monitoring of the three endocrine axes which are helpful for early detection are needed. Furthermore, long term corticosteroid therapy is recommended since low levels of ACTH or cortisol could maintain for a long period even though the clinical symptoms relieved, and it may contribute to a better outcome for allowing patients to continue ICIs therapy.

## Data Availability

All the data generated and/or analyzed during this study are included in this published article.

## Abbreviations

irAEs: Immune-related adverse events
ICIs: Immune checkpoint inhibitors
ACTH: Adrenocorticotropic hormone
IAD: Isolated ACTH deficiency
MRI: Magnetic resonance imaging
